# Correction: A transition-based neural framework for Chinese information extraction

**DOI:** 10.1371/journal.pone.0250519

**Published:** 2021-04-15

**Authors:** Wenzhi Huang, Junchi Zhang, Donghong Ji

Junchi Zhang should no longer be denoted as a corresponding author, and Donghong Ji should be denoted as the sole corresponding author. Donghong Ji’s email address is: dhji@whu.edu.cn.
